# Association between Anthropometric Measures and Indicators for Hypertension Control among Kazakh-Chinese Hypertension Patients in Xinjiang, China: Results from a Cross-sectional Study

**DOI:** 10.1371/journal.pone.0170959

**Published:** 2017-01-27

**Authors:** Qinghua Zhang, Tanmay Mahapatra, Feifei Huang, Weiming Tang, Yufang Guo, Songyuan Tang, Yang Lei, Lei Feng, Anni Wang, Liuyi Zhang, Jingping Zhang

**Affiliations:** 1 Xiang Ya Nursing School, Central South University, Changsha, Hunan, China; 2 School of Nursing, Xinjiang Medical University, Urumqi, Xinjiang, China; 3 University of California Los Angeles, Los Angeles, United States of America; 4 School of Nursing, Fujian Medical University, FuZhou, Fu Jian, China; 5 University of North Carolina Project-China, Guangzhou, China; 6 The Nethersole School of Nursing, The Chinese University of Hong Kong, Hong Kong, China; 7 Medical Department, The people’s hospital of Xinjiang Uyghur autonomous region, Urumqi, Xinjiang, China; The University of Tokyo, JAPAN

## Abstract

**Background:**

Among Kazakh-Chinese population in Xinjiang province of China, prevalence of obesity and hypertension were 40.1% and 50.3% respectively, the highest across all ethnic groups residing in this pastureland. Despite this, there remained a dearth of information regarding the association between the anthropometric measures [body mass index (BMI), waist circumference (WC), waist-hip ratio (WHR), Waist-to-height ratio (WHtR) etc.] and indicators for hypertension control [achieved levels of systolic and diastolic blood pressures (SBP and DBP), pulse pressure index (PPI), ankle-brachial index (ABI) etc.] among them.

**Method:**

A cross-sectional study was conducted in Xinjiang to determine the distribution and inter-relationships of the anthropometric measures and indicators for achieved BP control as well as their predictors among hypertension patients of Kazakh-Chinese ethnicity. Out of 550 randomly selected patients, 516 completed the interview, anthropometry and BP assessments.

**Results:**

In the sample population, average SBP, DBP and PP were 156.26±24.40mmHg, 87.55±14.73mmHg and 68.71±19.39mmHg respectively. Bivariate analysis identified age, gender, education, duration of hypertension, WC and BMI being factors influencing the achieved levels of BP. Adjusted multiple linear regression models elicited positive associations of age (β^a^ = 0.152, p = 0.001) and duration of hypertension (β^a^ = 0.132, p = 0.003) with achieved level of SBP as well as BMI (β^a^ = 0.135, p = 0.002) with DBP. Age (β^a^ = 0.207, p<0.001) and WHtR (β^a^ = 0.304, p = 0.005) were positively and WC (β^a^ = -0.406, p<0.001) was negatively associated with PPI. Increasing age (β^a^ = -0.125, p = 0.005), female gender (β^a^ = -0.122, p = 0.005) and 5years’ duration of hypertension (β^a^ = -0.091, p<0.039) were negatively associated with ABI. After adjustment for socio-demographic variables, hypertensive patients with (reference = without) abdominal obesity had 93% (p = 0.013) higher odds of missing the target BP control.

**Conclusion:**

Anthropometric measures and indicators for blood pressure control among Kazakh-Chinese patients were far beyond normal. Several anthropometric measures appeared useful for monitoring BP. Using them, regular screening and consequent targeted intervention were required urgently to control hypertension among Kazakh-Chinese.

## Introduction

Hypertension is regarded as one of the major contributing factor for the global burden of diseases[1]. Worldwide, two-third deaths from stroke and half of the deaths from coronary artery diseases are also attributed to hypertension[2]. In China, the prevalence of hypertension has increased from 19.8% in 2004 to 30.6% in 2010, and this alarming upsurge has been observed across age, gender and urban/rural areas[3]. In Xinjiang province, the scenario is even worse as evident from the observed hypertension prevalence of 40.1% (men: 42.1% vs women: 38.4%)[4]. In this province, persons belonging to Kazakh ethnicity have the highest prevalence of hypertension (50.3%), followed by Han (38.9%) and Uygur (32.9%) respectively[4]. Thus Hypertension has become one of the major public health concerns among Kazakh-Chinese people.

Pulse pressure index (PPI) is a relatively novel estimate of arterial stiffness defined as “pulse pressure/systolic pressure”[5]. The value of PPI ranges between 0 and 1. There is substantial evidence that PPI correlates negatively with vascular compliance[5]and can be considered an independent measure of cardiovascular mortality[6]. Prior research already demonstrated that hypertensive patients with higher PPI had a more reduced renal function[7]. Furthermore, PPI was also found to be useful in early screening of carotid atherosclerosis among these patients[8]. Despite this well-understood importance of PPI as a prognostic/screening indicator, in China, there remained a dearth of information regarding the burden of PPI among patients having hypertension, especially in Kazakh-Chinese community.

Ankle-brachial index (ABI) is another parameter having a strong correlation with cardiovascular adversities[9]. Since 2007, the guidelines for the management of arterial hypertension issued by the European Society of Cardiology (ESC) and European Society of Hypertension (ESH) did include ABI as a parameter for early detection of peripheral artery diseases(PAD)[10]. As per the reported evidences, ABI<0.9 also appeared to be an independent predictor of cardiovascular mortality[11].

Obesity has long been established as an important and independent risk factor for the development and complication of hypertension[12–14]. In China, the prevalence of obesity alarmingly increased from 3.75% in 1991 to 11.3% in 2011[15]. In Xinjiang, in 2010, this prevalence was 26.9%, while just like hypertension, Kazakhs had the highest burden (40.1%), followed by the Uyghurs (28.9%) and the Han (18.4%)[16]. Body Mass Index (BMI), Waist Circumference (WC), Waist-to-height ratio (WHtR) and Waist-hip ratio (WHR) are simple and valid anthropometric measures for the assessment of obesity and risk of hypertension[17, 18] However, the strengths of association of these four indexes with hypertension, PPI and ABI varied across population and those among Kazakh-Chinese hypertensives, were still unknown.

The aforementioned dearth of information regarding the distribution and determinants of indicators for blood pressure (BP) control and identification of appropriate anthropometric measures to monitor them, called for a detailed investigation involving the hypertensive patients of Kazakh-Chinese ethnic group.

## Methods

### Design

Between June 2013 and February 2014, a cross-sectional study was conducted to determine the distribution of the anthropometric measures (BMI, WC, WHR, WHtR etc.), indicators (SBP, DBP, PPI, ABI etc.) for achieved levels of BP control, their inter-relationship and predictors among Kazakh-Chinese hypertensive patients in Xinjiang pastureland in Western China. Using a stratified cluster random sampling strategy, hypertensive patients belonging to this minority group were recruited for the current study if they met all the following inclusion criteria: (a) systolic BP (SBP)≥140mmHg and/or diastolic BP (DBP)≥90mmHg or taking antihypertensive medication[19], (b) age≥18 years, (c) not suffering from any cognitive dysfunctions, severe enough to prevent appropriate communication and (d) provided voluntary informed consent for participating in the study. The cities/counties in Xinjiang province having congregation of Kazakh-Chinese communities were enlisted first and one of them was selected from the list randomly. Then hypertensive patients of Kazakh-Chinese ethnicity, aged≥18 years were identified based on individual health assessment records and periodic follow-up records of the residents, enlisted and then randomly recruited from that list according to their age and gender distribution.

### Sample size

Information on SBP level (158±22 mmHg) among Kazakh-Chinese hypertensive patients measured in an epidemiological investigation in Xinjiang during 2010[20], was used as the parameter value for the sample size calculation. Using the formula N = (1.96*S/δ)^2^[21], where S = standard deviation and δ = allowable error, assuming α = 0.05, the desired sample size (N) was determined to be 465 patients [(1.96*22/2)^2^]. Further assuming 20% non-response 550 eligible subjects were required to be recruited for the study.

### Recruitment

Investigators and community public health physicians conducted the face-to-face interview and measurements (BP and anthropometry) respectively at home or health service centers.

### Ethics statement

Prior to the interview and examinations, written informed consent was obtained from each participant after explaining all details pertaining to the study. The content and procedure of the study were reviewed and approved (Reference No: 20130216–134) by the Ethics Committee of the First Affiliated Hospital of Xinjiang Medical University, Xinjiang, China.

### Data collection

Using a pre-tested, structured, questionnaire, face to face interviews with eligible participants were conducted to collect information on the socio-demographic and behavioral aspects followed by the physical assessment and anthropometry.

### BP and anthropometric measurements

After allowing the subject to rest for 5–10 minutes, two arm-type electronic sphygmomanometers (UA-621, A&D Medical, Japan)[22] were used to measure the right brachial BP at the level of the heart and the corresponding right ankle BP at the medial point between the external ankle and the malleolus in supine position.

Standardized techniques and calibrated devices were used for all anthropometric measurements. For each of these measurements, arithmetic average of the observed values from two repeated observations were used. Height (in cm) was measured and rounded to the nearest 0.1 cm, using a stadiometer, with participants standing upright, without shoes. Body weight (kg) was measured and similarly rounded to the nearest 0.1 kg using an automatic electronic scale, with participants wearing light clothing and not wearing footwear. WC (in cm) was measured to the nearest 0.1cm, by an anthropometric tape, at the midpoint between the last palpable rib and iliac crest while the participants stood with feet 25–30 cm apart.

### Definitions and criteria

According to the JNC-8 the 2014 guideline, the targets for the general population aged≥60 years were defined as: “SBP<150mmHg and DBP<90mmHg”[23]. In the general population aged<60 years, SBP<140mmHg and DBP<90mmHg were targeted. In the population aged ≥18 years with diabetes or CKD, targets were set at: “SBP<140mmHg and goal DBP<90mmHg[23]” ABI was calculated as the systolic BP measured at ankle divided by that in brachial. ABI<0.9 an identified predictor for cardiac complications, was defined as the diagnostic criteria for the peripheral arterial disease [[24]]. Based on BMI [weight in kg/(height in meter)^2^] participants were categorized into three groups: obese (BMI ≥30), overweight (25≤BMI< 30) and normal/ underweight (BMI<25). Abdominal obesity was determined based on both WC as well as Waist-to-hip ratio (WHR = WC in cm/hip circumference in cm). Men with WC≥102cm and women with WC≥88cm were considered as having abdominal obesity while abdominal overweight meant 94cm≤WC<102cm in men and 80cm≤WC< 88cm in women. WC<94cm in men and WC<80cm in women were considered normal[25]. Again WHR≥0.90 in man and WHR≥0.85 in women were classified as abdominal obesity[26, 27]. Additionally, Waist-to-height ratio (WHtR = WC in cm/height in cm) ≥0.5 was defined as centralized obesity[28].

### Data analysis

All analyses were conducted using SPSS version 20.0 (S1 Dataset). The results were considered statistically significant when p value was < 0.05. Distributions of the participant characteristics and anthropometric findings were determined by descriptive analyses [mean±standard deviation (SD) for continuous and subgroup-specific percentages for categorical variables]. T-test of Independent sample and One-way ANOVA were used to compare the continuous variables (SBP, DBP, PPI, ABI, BMI, WC, WHR and WHtR). Associations between categorical variables were tested using Chi-square test. Bivariate analysis was used to explore the association between BP and anthropometric findings. Multiple stepwise regression analysis was used to determine the risk factors for SBP, DBP, PPI and ABI among participants. To understand the role of the potential determinants in achieving targeted level of control over BP, binary logistic regression analysis was performed adjusting for age, gender and medical treatment.

## Results

### Sample characteristics

Among 550 randomly selected eligible subjects, 516 (46.3% males and 53.7% females) completed the interview along with the clinical examination and anthropometric measurements. The overall response rate was 93.82%. The mean age was 58.14±12.05 years. Mean BMI was found to be 27.77±4.80 and mean WC was 97.56±13.07. Only 34.5% were receiving regular medical treatment for hypertension at the time of the survey. BP of 72.7% patients never reached the target level. Among participating patients, an average SBP was 156.26±24.40mmHg, DBP was 87.55±14.73mmHg and the PP was 68.71±19.39mmHg. (Table 1)

**Table 1:** Characteristics of the Kazakh-Chinese hypertension patients (N = 516) residing in the pasture area of Xinjiang, China, 2014.

Numerical variables	Mean±SD
**Demographic characteristics**	
Age(years)	58.14±12.05
**Anthropometric characteristics**	
Blood pressure (BP, mmHg)	
SBP	156.26±24.40
DBP	87.55±14.73
PP	68.71±19.39
ABI	1.14±0.14
BMI	27.77±4.80
WC	97.56±13.07
WHR	0.95±0.09
WHtR	0.60±0.09
Categorical variables	Frequency (%)
**Demographic characteristics**	
Gender	
Male	239(46.3)
Female	277(53.7)
Education level	
Less than high school	359(69.6)
High school or higher	157(30.4)
Marital status	
Married	431(83.5)
Unmarried/ Divorce/Widowed	85(16.5)
Occupational status	
Unemployed	183(35.5)
Part-time employment	83(16.1)
Full-time employee	250(48.4)
Family annual income	
<10,000RMB	390(75.6)
10,000~30,000RMB	103(20.0)
≥30,000RMB	23(4.5)
Family history of hypertension(years)	
No	215(41.7)
Yes	301(58.3)
Complication of hypertension	
No	422(81.8)
Yes	94(18.2)
Years with hypertension (years)	
<5	332(64.3)
≥5	184 (35.7)
Medication treatment	
No	338(65.5)
Yes	178(34.5)

In addition, 15 individuals suffering from diabetes, 6 individuals had chronic kidney disease, 64 had coronary heart disease, 7 suffered from cerebral stroke, 3 individuals had both diabetes and coronary heart disease while 2 individuals had cerebral stroke combined with coronary heart disease.

### Distribution of BP-related measures across socio-demographic strata

Older age groups had higher SBP than <50years aged (156.60±26.67 for 50–60 years, 156.77±22.42 for 60–70 years and 162.38±24.12 for >70 years vs. 150.59±23.19mmHg for <50 years, p<0.05). Again 60–70 years old patients had lower DBP than 50–60 years’ group (85.41±13.29 vs. 90.50±17.26mmHg, p<0.01). PPI was increasing, but ABI was decreasing with advancing age. SBP and PPI of females were higher than males (SBP: 158.30±25.29 vs. 153.88±23.14mmHg, and PPI: 0.44±0.08 vs. 0.43±0.08mmHg, p<0.05). BP control was marginally better among men than women (28.0% vs. 26.7%). Patients who did not attend high school had higher SBP than those who had high school or higher level of education (157.65±24.24 vs. 153.06±24.53 mmHg, p<0.05). SBP and PPI of full-time employed subjects were higher than the part-time workers (SBP: 156.08±24.76 vs. 148.55±23.08 mmHg, and PPI: 0.43±0.08 vs. 0.40±0.08mmHg, p<0.05). Those who were having hypertension for ≥5years had higher SBP and ABI than those suffering from <5 years (SBP: 161.55±24.97 vs.153.32±23.60 mmHg, and ABI: 1.15±0.14 vs.1.12±0.13, p<0.01). SBP, DBP, PPI, and ABI did not vary significantly across the categories of complication and treatment of hypertension. Also, there was no statistical significance for the variation in the proportion of patients for whom targeted control of BP across the strata of received treatment (p = 0.756). (Table 2)

**Table 2:** Distribution of blood pressure related measures across the socio-demographic strata among Kazakh-Chinese hypertension patients (N = 516) residing in the pasture area of Xinjiang, China, 2014.

Variables	n	SBP (mmHg)	DBP (mmHg)	PPI	ABI
Age(years)					
<50	130	150.59±23.19	87.46±13.68	0.41±0.08	1.17±0.13
50~60	139	156.60±26.67*	90.50±17.26	0.42±0.08	1.14±0.13
60~70	147	156.77±22.42*	85.41±13.29^△△^	0.45±0.08**^△^^△^	1.13±0.14*
≥70	100	162.38±24.12**	86.71±13.74^△^	0.46±0.08**^△△^	1.10±0.12**^△^^Ϯ^
Gender					
Male	239	153.88±23.14	87.49±14.88	0.43±0.08	1.16±0.14
Female	277	158.30±25.29*	87.60±14.62	0.44±0.08*	1.12±0.13**
Education					
Less than high school	359	157.65±24.24	87.80±14.62	0.44±0.08	1.14±0.13
High school or higher	157	153.06±24.53*	86.98±14.99	0.43±0.07	1.14±0.15
Complication of hypertension					
No	299	155.61±24.42	87.41±14.90	0.43±0.08	1.14±0.14
Yes	85	159.14±24.22	88.17±13.98	0.44±0.07	1.12±0.13
Years of hypertension(years)					
<5	332	153.32±23.60	88.84±14.11	0.43±0.08	1.15±0.14
≥5	184	161.55±24.97**	88.83±15.74	0.45±0.08*	1.12±0.13**
Medication treatment					
No	338	155.07±24.85	86.64±15.67	0.44±0.08	1.14±0.14
Yes	178	158.51±23.41	89.26±12.62	0.43±0.07	1.14±0.13

*P < 0.05.

**P < 0.01 in comparison with age <50, male, less than high school, years of hypertension<5year.

^△^*P* < 0.05.

^△△^*P* < 0.01 in comparison with age 50~60.

^Ϯ^ *P* < 0.05 in comparison with age 60~70.

### Distribution of BP-related measures across categories of anthropometric findings

Compared to those with BMI<25kg/m^2^, obese subjects had higher DBP and lower PPI (p<0.01). SBP and DBP were increasing with increase in WC, WHR and WHtR. Compared those with normal WC, patients having abdominal obesity (based on WC) had higher SBP and DBP (SBP: 157.72±24.04, vs. 152.44±23.98, p<0.05 and DBP: 88.93±13.96 vs. 84.69±15.99mmHg; p<0.01). (Table 3)

**Table 3:** Distribution of the BP-related measures across the strata of anthropometric findings among Kazakh-Chinese hypertension patients (N = 516) residing in the pasture area of Xinjiang, China, 2014.

Variables	n	SBP (mmHg)	DBP (mmHg)	PPI	ABI
*BMI*					
*BMI<25*	151	155.72±25.25	86.75±16.70	0.44±0.07	1.12±0.15
*25≤BMI<30*	213	155.39±24.11	85.15±11.93	0.45±0.08	1.15±0.14
*BMI≥30*	152	158.00±24.00	91.70±15.38**^△^	0.41±0.09**^△^	1.14±0.12
**WC**					
*<94cm(men)*	112	152.44±23.98	84.69±15.99	0.44±0.08	1.14±0.16
*<80cm(women)*					
*94≤WC<102cm(men)*	85	155.79±25.95	86.13±15.29	0.44±0.08	1.15±0.12
*80≤WC<88cm(women)*					
*≥102cm(men)*	319	157.72±24.04**	88.93±13.96**	0.43±0.08	1.14±0.13
*≥88cm(women)*					
**WHR**					
<0.90(men)	83	154.10±25.21	86.98±18.62	0.43±0.07	1.15±0.16
< 0.85(women)					
≥0.90(men)	433	156.67±24.24	87.66±13.88	0.43±0.08	1.14±0.13
≥0.85(women)					
**WHtR**					
<0.5	62	152.15±23.73	84.42±13.87	0.44±0.08	1.14±0.16
≥0.5	454	156.82±24.46	87.98±14.80	0.43±0.08	1.14±0.13

*P < 0.05.

**P < 0.01 in comparison with BMI <25; WC<94cm in men and 80cm in women.

^△^*P* < 0.01 in comparison with 25≤BMI<30.

### Factors influencing BP-related measures

After adjusting for treatment, socio-demographic and anthropometric correlates of SBP, DBP, PPI, and ABI were determined through multiple linear regression modelling. Age (adjusted coefficient: β^a^ = 0.152, p = 0.001) and duration of hypertension (β^a^ = 0.132, p = 0.003) had positive associations with SBP. Patients with higher BMI had more DBP (β^a^ = 0.135, p = 0.002). PPI was associated negatively with WC (β^a^ = -0.406, p<0.001) and positively with age (β^a^ = 0.207, p<0.001) and WHtR (β^a^ = 0.304, p = 0.005). Age (β^a^ = -0.125, p = 0.005), gender (β^a^ = -0.122, p = 0.005) and duration of hypertension (β^a^ = -0.091, p = 0.039) were negatively associated significantly with ABI. (Table 4)

**Table 4:** Significant correlates of SBP, DBP, PPI and ABI among Kazakh-Chinese hypertension patients (N = 516) residing in the pasture area of Xinjiang, China, 2014.

Risk factors	B	Std. Error	B^a^	*t*	*P* value
**SBP**					
Age	0.307	0.089	0.152	3.455	0.001
Years of hypertension (years)	6.718	2.235	0.132	3.006	0.003
**DBP**					
BMI	0.415	0.134	0.135	3.095	0.002
**PPI**					
Age	0.001	0.000	0.207	4.657	<0.001
WC	-0.002	0.001	-0.406	-3.805	<0.001
WHtR	0.272	0.096	0.304	2.828	0.005
**ABI**					
Age	-0.001	0.000	-0.125	-2.840	0.005
Gender	-0.033	0.012	-0.122	-2.815	0.005
Years of hypertension (years)	-0.026	0.012	-0.091	-2.066	0.039

^a^**Adjusted for Medication treatment.**

### Anthropometric findings and achieved level of BP through control measures

After adjustment for age, gender, duration and treatment of hypertension, abdominal obesity (OR: 1.932; 95%CI: 1.149~3.249; p = 0.013) was found to be associated positively with achieved BP levels through control measures (Table 5).

**Table 5:** Association between waist circumference and BP control among Kazakh-Chinese hypertension patients (N = 516) residing in the pasture area of Xinjiang, China, 2014.

Risk factors	Reference	B^a^	Odds ratio	95% Confidence interval	*P* value
94≤WC<102cm(men)	<94cm(men)	0.025	1.025	0.561~1.874	0.935
80≤WC<88cm(women)	<80cm(women)				
WC≥102cm(men)	<94cm(men)	0.658	1.932	1.149~3.249	0.013
WC ≥88cm(women)	<80cm(women)				

^a^ **Standardized coefficients.**

Adjusted for Age, Gender, Years of hypertension, and Medication treatment.

## Discussion and Conclusion

Kazakh-Chinese community is the second largest ethnic minority group in Xinjiang. They live in the mountains, eat cheese and meat more with less intake of vegetables and fruits. Such unhealthy food habit was likely to increase the risk of obesity, hyperlipidemia and hypertension in this population. Thus it was not surprising that among Kazakh-Chinese residents of Xinjiang province, based on prior findings, 50.3% were suffering from hypertension[4] and 40.1% from obesity[16], highest among any other ethnic groups in this province.

In the current study, also, the observed burden of general (29.46%) and abdominal obesity (61.82%) were very high among Kazakhs. Corroborating with several prior studies, anthropometric measures like BMI, WC, WHR and WHtR were positively associated with achieved BP level. Generally, the predictive roles of anthropometric indicators in BP control vary across population. Regzedmaa et al[29] noted that as opposed to WHR, BMI was more consistent while predicting the incidence of hypertension in Mauritian Indian women. On the other hand, Bektas et al. [30] observed that WC had stronger positive association with elevated BP. As a part of the hypertension control program in the Kazakh pastureland, the current study aimed to identify the anthropometric measures corroborating strongly with achieving hypertension control among Kazakh-Chinese residents of Xinjiang, China. Other objectives included identification of the important predictors for hypertension control (through achieving target levels of BP, SBP, DBP, PPI and ABI) among hypertensive patients in Kazakh community.

Growing bodies of evidence suggested elevated SBP to be a strong and direct predictor for mortality among hypertensive patients aged >50 years[31, 32]. In this study the highest SBP (162.38 ±24.12mmHg) was observed among patients aged >70 years. While further analysis through multiple stepwise regression identified age and duration of hypertension as strong correlates of SBP, the anthropometric measures failed to satisfy the model inclusion criteria.

High DBP had been found previously to be associated with higher likelihood of organ damage among patients aged <50 years[31, 33]. In this study, DBP was highest (90.50±17.26 mmHg) among patients aged 50–60 years. Multiple stepwise regression analysis identified a significant association between BMI and DBP. Based on the findings, it appeared that screening and monitoring of BMI should be made an integral part of the hypertension control program in this pastureland especially targeting the Kazakh-Chinese patients having uncontrolled DBP.

It was also revealed that SBP, DBP and PPI were higher among women than men, while the case was reverse for ABI. Thus, contradicting with some prior observations[34], this study indicated relatively poorer control of BP among Kazakh women compared to their male counterparts. The observation was supported by the findings that the prevalence of obesity and abdominal obesity were also significantly high among women than men (32.9% vs. 25.5% and 79.8% vs. 41.0%) in this study. Potential explanations included the gender differences in lifestyle of the Kazakhs, especially among those who adhered to traditional nomadic life, residing in remote mountains. These men were very active, involved in physically demanding, outdoor jobs like taking care of livestock in hilly terrains, whereas women remained less physically active, busy in household works and child-care.

Determination of PPI, through BP measurement, was found to be rapid. Previous research also established it as a better indicator than PP for the assessment of cardiovascular outcomes[5]. To the best of our knowledge, this was the first effort to deduce correlation between PPI and the anthropometric indicators. Bivariate analysis revealed that females, those aged >60 years, having full-time occupation, suffering from hypertension for >5 years, and having 25≤BMI<30 were more likely to have higher PPI. Multiple stepwise regression analysis indicated that age and WHtR were positively and WC was negatively associated with PPI. Earlier studies did also indicate WHtR as a better correlate of hypertension compared to BMI and WC among adolescents[35], children[36] and large (>3000,000) population in general[37]. Explanations included the ability of WHtR to rule out the influence of height resulting into better correlation with cardio-metabolic risk among majority irrespective of their heights. Hence it appeared that for achieving better control of hypertension and thus higher life expectancy in this Kazakh-Chinese community, public health professionals should emphasize on WHtR instead of WC among patients and warrant them to “keep waist circumference below half of the height”.

ABI measurement had widely been recommended for PAD screening[38]. Abnormal ABI (<0.9 or >1.3) was found to increase the risk of cardiovascular death among hypertensive patients by 3–5%[39]. In the current study, 2.9% hypertensive patients had ABI<0.9 while for 11.2% it was≥1.3. Alike elsewhere[40], females and those suffering longer (≥5 years) from hypertension had higher likelihood of having abnormal ABI.

After adjustment for age, gender, duration of hypertension and medication, hypertensive patients having abdominal obesity were 1.9 times likely to fail to achieve the target BP through the existing control program. Thus it appeared that prior screening and monitoring of WC might help in identification and then targeted intensification of intervention among patients having abdominal obesity, to improve their chances of achieving BP control.

The study had some important limitations. First, hypertensive patients of Kazakh ethnicity were recruited for this study through a community-based sampling which could have missed some patients not residing locally for study (younger ones) or jobs elsewhere. Thus, further efforts should be made using multiple strategies to make the sample more comprehensive in future. Non-inclusion of any healthy control group could also be viewed as another limitation of this study that involved only the hypertensive patients. Besides, in order to ensure the reliability, 34 subjects with incomplete data were excluded, which might have limited the generalizability of the findings of this study to some extent in addition to the age-related issue. Alike any observational study with cross-sectional design, the observed associations should not be interpreted as causal and any effort to extrapolate the results beyond the study sample to be made with caution. Owing to the very small number of diagnosed cases of non-communicable diseases in the study sample, determination of the association between anthropometric measures and BP control among those specific cases was not possible. Last but not the least, our study did not collect detailed information on high blood pressure treatment.

## Conclusions

Observed values for the hypertension related indicators including SBP, DBP, PP and PPI as well as anthropometric measures like BMI, WC, WHR and WHtR were far beyond the normal ranges among hypertensive patients of Kazakh-Chinese ethnicity in Xinjiang pastureland. Subjects having higher anthropometric measures like BMI, WC, WHR, and WHtR, were more likely to end up with higher achieved levels of SBP, DBP, PP and PPI through efforts for hypertension control. After adjustment for the potential socio-demographic confounders, BMI and WHtR appeared to be independent risk factor for having higher DBP and PPI levels respectively. WC was also found to be associated positively with odds of having uncontrolled hypertension among Kazakh-Chinese residents of the study area. Thus for better effectiveness, hypertension control programs in this part of China, need to include anthropometric screening and targeted intervention for the identified population susceptible for poor BP control.

## Supporting Information

S1 Dataset (SAV)
